# Trimetallic PdCuAu Nanoparticles for Temperature Sensing and Fluorescence Detection of H_*2*_O_*2*_ and Glucose

**DOI:** 10.3389/fchem.2020.00244

**Published:** 2020-04-07

**Authors:** Furong Nie, Lu Ga, Jun Ai, Yong Wang

**Affiliations:** ^1^College of Chemistry and Enviromental Science, Inner Mongolia Normal University, Hohhot, China; ^2^College of Pharmacy, Inner Mongolia Medical University, Hohhot, China; ^3^College of Geographical Science, Inner Mongolia Normal University, Hohhot, China

**Keywords:** trimetallic alloyed nanoparticles, temperature-sensitive, sensor, peroxidase-like activity, colorimetric system

## Abstract

The design of palladium-based nanostructures has good prospects in various applications. This paper reports a simple one-step synthesis method of PdCuAu nanoparticles (PdCuAu NPs) prepared directly in aqueous solution. PdCuAu NPs have attracted much attention owing to their unique synergistic electronic effect, optical and catalytic performance. As temperature sensor, PdCuAu NPs are sensitive to the fluorescence intensity change in the temperature range of 4–95°C, which is due to its unique optical properties. The prepared PdCuAu NPs have excellent catalytic performance for peroxidase-like enzymes. It can catalyze TMB rapidly in the presence of hydrogen peroxide and oxidize it to visible blue product (oxTMB). Based on its unique peroxidase-like properties, this study used PdCuAu NPs colorimetric platform detection of hydrogen peroxide and glucose. The linear ranges of hydrogen peroxide and glucose were 0.1–300 μM and 0.5–500 μM, respectively, and the detection limits (LOD) were 5 and 25 nM, respectively. This simple and rapid method provides a good prospect for the detection of H_2_O_2_ and glucose in practical applications.

In recent years, the research on the preparation and application of nano-device mainly focuses on the preparation and application of polymetallic nanomaterials (Dubau et al., 2015; Xia et al., 2015; Luo and Shen, 2017; Yang et al., 2017; Tang et al., 2018). Because of their unique physical and chemical properties, polymetallic nanomaterials have been widely used in optics, chemical reactions, fuel cells, sensors and catalysts (Huang et al., 2014; Khan et al., 2015; Tang et al., 2018; Wang et al., 2018). At present, Pt-based nanomaterials are the most widely used catalysts because of their high catalytic activity in cathodic and anodic reactions (Khan et al., 2015; Wang et al., 2018). Consequently, tri-metallic Pt-based alloys for instance Pt-Ni-Cu (Tang et al., 2018), Pt-Ni-Co (Huang et al., 2014; Sriphathoorat et al., 2016), Pt-Pd-Cu (Yin et al., 2012; Tian et al., 2017), Pt-Pd-Co (Cho et al., 2014; Song et al., 2015), Pt-Fe-Ni (Li and Chan, 2013), Pt-Fe-Co (Hwang et al., 2011), and Pt-Pd-Au (Li et al., 2018) aroused the attention of many studies. However, due to the low cost and high catalytic activity of Pd-based catalysts, there are few studies on Pd-based catalysts (Guo et al., 2014; Jiang et al., 2015; Xu et al., 2015; Yousaf et al., 2017a), therefore, Pd-based catalysts are the most promising substitute for Pt-based catalysts (Xu et al., 2015; Yousaf et al., 2017a; Wang et al., 2018). Many researchers have done a lot of work on Pd-based catalysts to improve the catalytic performance of palladium catalysts (Wang X. et al., 2015; Xue et al., 2017; Yousaf et al., 2017b). Wang et al. reported a very simple method to synthesize trimetal PdCuAu nanoparticles (NPs) with branched structure, so the PdCuAu NPs have excellent catalytic performance, durability and methanol oxidation resistance (Wang et al., 2018). Huang et al. proposed to prepare an ordered PdCu-based NPs (PdCuCo, and PdCuNi) by colloidal chemistry. PdCuCo NPs have excellent stability and activity in redox reaction because of their intermetallic phase and composition advantages (Jiang et al., 2016). Yang's group reported that the CuPd alloy with controllable shape was preparation of oleylamine by electric substitution reaction. Put Cu in Pd could reduce the bond intensity among the intermediate and palladium, cause the enhancement of lattice shrinkage, thus improving the electrocatalytic performance of redox reaction (Chen D. et al., 2017). Consequently, the Pd-based alloy nanomaterials are considered as a promising catalyst with enhanced catalytic performance. However, Pd-based alloy nanomaterials are seldom used in the field of sensors. In this paper, the application of Pd-based alloy nanomaterials in sensors is studied.

In recent years, thermosensitive materials have shown significant activity due to their possible applications in nanoscale temperature measurement (Zhou et al., 2018). Though, many temperature sensitive materials have a single signal response, and the fluorescence stability and contrast are poor. Dong group studied a novel dual fluorescence temperature sensor based on DNA- template Ag NCs. It has two fluorescence peaks and can be used for sensitive detection of temperature changes from 15 to 45°C (Zhou et al., 2018). Oemrawsingh studied that the single emitter fluorescence of Ag NCs increased 5-fold when the temperature dropped from 295 to 1.7 K (Oemrawsingh et al., 2012). Chen et al. reported a hairpin-like Ag NCs with DNA template. The Ag NCs exhibited reversible fluorescence properties between 25 and 66°C due to the loosening and compacting of the four-stranded template structure (Zhao et al., 2015). A one-step synthesis method for prepared high fluorescence bimetallic Cu-Au nanoclusters (Cu/Au BNCs) was proposed by Ai group. The fluorescence signals of Cu/Au BNCs exhibited reversible response and good sensitivity in the temperature range of 20–70°C (Nie et al., 2018). The Pd-based alloy nanomaterials have been successfully applied to temperature sensing in this paper.

Peroxidases are an important biocatalyst in organism, which can catalyze many kinds of biochemical reactions effectively, for example, they can deactivate toxic substances, oxidize fatty acids, regulate oxygen concentration and so on. Because of their incredible efficiency and high substrate specificity, they are of great importance in diagnosis and analysis (Guo et al., 2018). In recent years, nanozymes have attracted much attention due to their endogenous mimic enzymes similar to natural enzymes, it can catalyze substrate reaction (Yan et al., 2018). Compared with natural enzymes, nano-enzyme production process is simple and economical, and it has excellent robustness and stability. In the past, researchers have found that various nanomaterials possess catalytic properties of peroxidase-like enzymes. Among many nanomaterials, inorganic nanomaterials have also attracted attention, including metal oxides V_2_O_3_ (Han et al., 2015), NiO (Liu et al., 2015), CuO (Wang et al., 2013; Chen M. et al., 2017), CeO_2_ (Liu et al., 2017a; Sun et al., 2017; Ge et al., 2019; Yang et al., 2019), and sulfides ZnS (Liu et al., 2017b), CdS (Liu et al., 2014), CuS (Zhang et al., 2017a) etc. Compared to the analytical methods including electrochemistry (Niu et al., 2013), fluorescence (Hu et al., 2014; Shan et al., 2014), chemiluminescence (Luo et al., 2015), mass spectroscopy (Chen et al., 2012), colorimetric detection method has the advantages of low cost, high selectivity and strong practicability, and is favored by researchers. In addition, because the color change of the substrate does not require any complicated instrument, it is easy to observe with the naked eye, so it has a wide range of applications in many fields (Ding et al., 2017).

Herein, we report a one-step synthesis of trimetallic PdCuAu nanoparticles (NPs) in aqueous phase without any intermediates. The sensitive fluorescence signals of PdCuAu NPs are reversible and recyclable in the range of 4–95°C. The prepared PdCuAu NPs show superior catalytic activity and sensitive in answer to the chromogenic substrate TMB, it can catalyze TMB in the presence of H_2_O_2_. So, the flow chart for the preparation of PdCuAu NPs and the application of PdCuAu NPs as temperature sensors and peroxidase-like enzymatic reactions are described in Scheme 1.

**Scheme 1:**
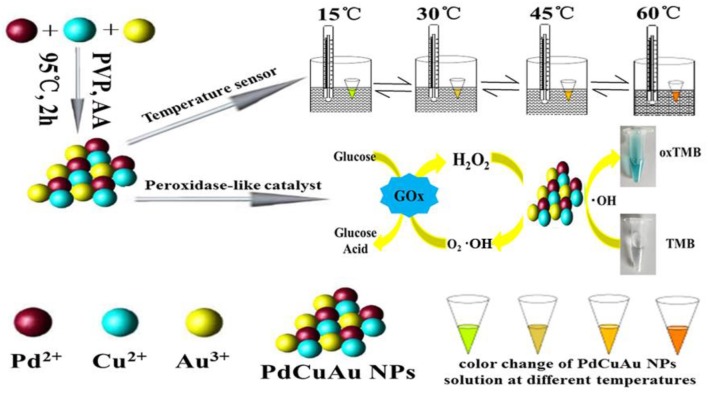
Synthesis of PdCuAu NPs and their application as temperature sensors and colorimetric detection of H_2_O_2_ and glucose with enzyme mimic.

## Results and Discussion

### Optimization and Characterization of the Synthesis Conditions of PdCuAu NPs

This is a very simple and easy to operate synthesis process: take 355 μ L H_2_PdCl_4_ solution (56.4 mm), 1 ml Cu (NO_3_)_2_ solution (0.1 M), and 412 μ L HAuCl_4_ solution (48.6 mM) and mix them evenly, and then add 500 μ L HCl (10%), 100 mg KBr and 50 mg PVP into them after ultrasonic degradation, and then add 2 ml ascorbic acid (AA) (0.1 M) After heating the mixed solution in a water bath at 95°C and stirring for 2 h, the primary product PdCuAu NPs is obtained after natural cooling. The primary product PdCuAu NPs is centrifuged at a centrifugal rate of 5,000 rpm for 15 min, and then the supernatant and sediment are centrifuged. The purified final product is stored in 4 In the environment of °C, it is used for the following analysis and characterization experiments, i.e., ultraviolet spectrum analysis, fluorescence spectrum analysis, transmission electron microscopy test, infrared spectrum test, XRD and XPS test.

**Figure 1:**
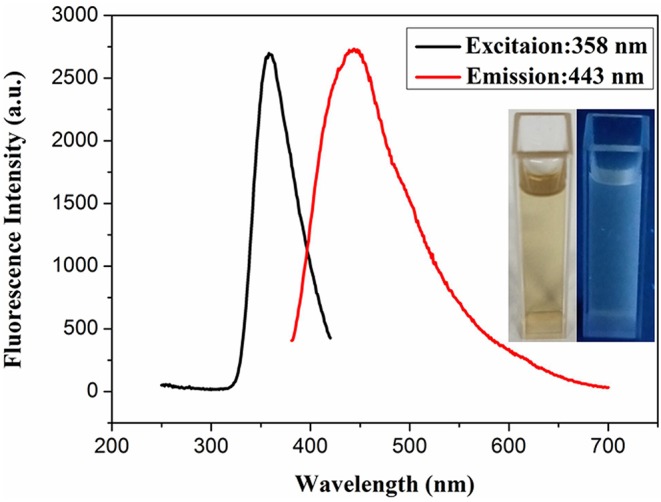
Fluorescence spectra of PdCuAu NPs. [Insets, the left panel was shown in visible light, and the right was viewed under UV radiation (365 nm)].

**Figure 2:**
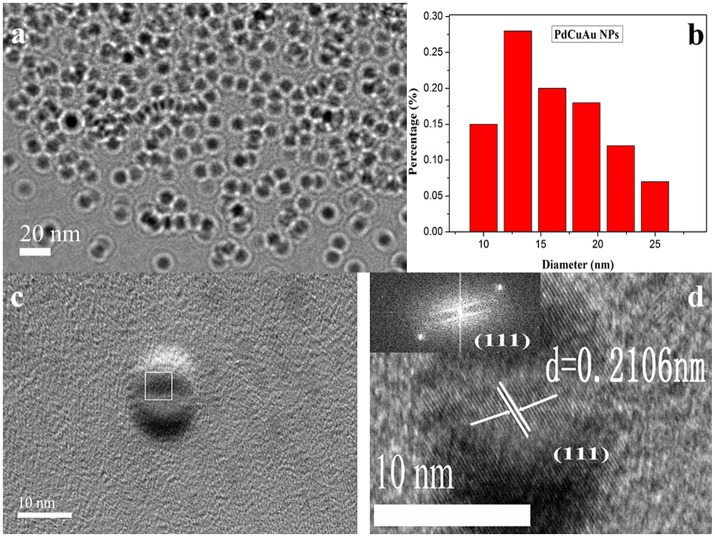
**(a)** TEM images of the PdCuAu NPs. **(b)** The size distribution of PdCuAu NPs. **(c)** HRTEM image of the PdCuAu NPs. **(d)** The lattice fringes in the square area in **(c)** and the inset displays the corresponding FFT pattern.

The reaction mixture was heated for 2 h by simple heat treatment at 95°C in a water bath. The obtained tri-metallic PdCuAu NPs were canary yellow in water phase (see Figure 1, inset, left). The pale blue fluorescence was observed in the PdCuAu NPs solution under UV illumination at 365 nm (see Figure 1, inset, right), and the PdCuAu NPs are excited at 358 nm and emit at 443 nm. Typical magnification transmission electron microscopy (TEM) and high-resolution TEM (HRTEM) images of the as-prepared product are shown in Figure 2. Their particles are distributed between 10 and 25 nm, with an average particle size of 13 nm (see Figures 2a,b). Since the measured lattice distance of PdCuAu NPs are 0.2106 nm, the lattice fringes of PdCuAu NPs are assigned to the (111) plane of the Fourier filtering image (see Figures 2c,d).

**Figure 3:**
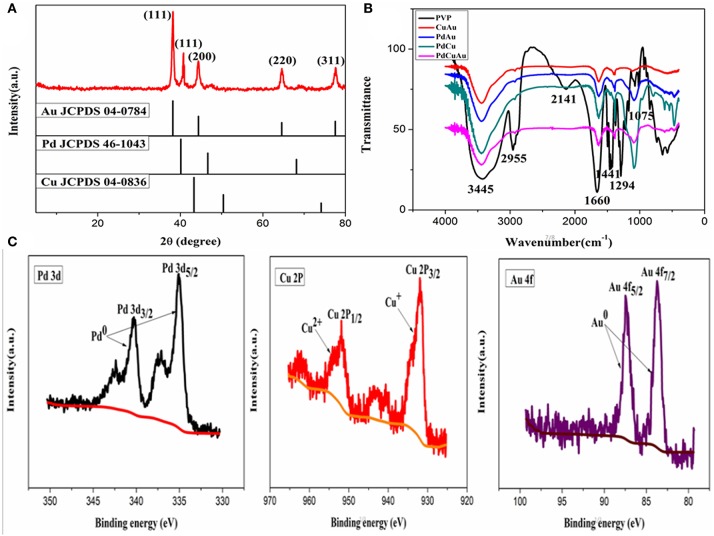
**(A)** XRD pattern of PdCuAu NPs, the standard patterns of pure Au (JCPDS card no. 04-0784), Pd (JCPDS card no. 46-1043) and Cu (JCPDS card no. 04-0836) are shown for comparison. **(B)** FT-IR spectra of pure PVP, CuAu, PdAu, PdCu, and PdCuAu NPs. **(C)** X-ray photoelectron spectroscopy spectra of Pd 3d, Cu 2p, and Au 4f of the as-prepared PdCuAu NPs.

The crystal structure of PdCuAu NPs is measured by XRD. As shown in Figure 3A, the representative diffraction peaks at 38.18, 40.12, 44.39, 64.58, and 77.55° are assigned to the (111), (111), (200), (220), and (311) planes of PdCuAu NPs, respectively. The peak positions of PdCuAu NPs are all situated between the homologous peaks of pure Au (JCPDS card no. 04-0784) and Pd (JCPDS card no. 46-1043), which confirm the formation of PdCuAu alloy. Both the HRTEM pattern (see Figures 2c,d) and from the XRD analysis (see Figure 3A), we can see that PdCuAu NPs show good crystallinity. The surface properties of PVP and CuAu, PdAu, PdCu, and PdCuAu NPs were characterized by FT-IR spectrometer. Exactly as shown in Figure 3B, the peak at 3,445 and 1,075 cm^−1^ were the characteristic absorption peak of the N-H stretching vibration of PVP, 2,955 cm^−1^ for the C-H stretching vibration of aromatic ring, 2,141 cm^−1^ for the stretching vibration of C-H outside the surface, 1,660 and 1,441 cm^−1^ for the C-C stretching vibration of aromatic ring skeleton, 1,294 cm^−1^ for the C-N stretching vibration of aromatic hydrocarbons. By comparison, the peak at 2,955 and 2,141 cm^−1^ disappeared in PdCuAu NPs, it indicates the breakup of C-H bond and the formation of new copper compounds. As determined by the TEM-EDS analysis, the atomic percentage of Pd in these nanocrystals is 42.14% and Au in these nanocrystals is 55.62%, the atomic ratio of Pd/Au in the as-prepared sample is ~1:1 (see Figure S1). Among the TEM-EDS pattern (see Figure S1), FT-IR pattern (see Figure 3B) and XPS pattern (see Figure 3C) show that the formation of copper compounds. The surface chemical composition and valence state of PdCuAu NPs were further studied by X-ray photoelectron spectroscopy (XPS). As shown in Figure 3C, the Pd 3d region of the sample can be divided into two pairs of doublets. Two Pd 3d peaks are located at 335.6 and 340.9 eV, corresponding to the Pd 3d_5/2_ and Pd 3d_3/2_ states of metallic Pd, respectively. The binding energies at 932.4 and 952.3 eV correspond to Cu 2p_3/2_ and Cu 2p_1/2_ by fitting these peaks, which are assigned to Cu^+^ and Cu^2+^, respectively. Similarly, the peaks at 83.8 and 87.5 eV are attributed to Au 4f_7/2_ and Au 4f_5/2_ by fitting these peaks, it is proved that Au^3+^ is reduced to Au^0^. Therefore, Pd and Au are the main species of PdCuAu NPs, so PdCuAu NPs have the potential to be effective catalysts.

Some controlled contrast experiments were carried out to explore the influencing factors in the process of synthesizing PdCuAu NPs. Pd/Cu/Au molar ratio, (Pd/Cu/Au = 1:1:1, 1:2:1, 1:1:2 mol/mol) were researched and the corresponding fluorescence spectra were shown in Figure S2A. The optimal Pd/Cu/Au molar ratio is 1:5:1, the fluorescence intensity of PdCuAu NPs (1:5:1) are the strongest (see Figure S2B) and it can also be obtained from the TEM image that the dispersion of PdCuAu NPs (1:5:1) are the best and the particle size is uniformity (see Figure S3). Figure S4 showed that the PdCuAu NPs exhibited the maximum fluorescence intensity under a water bath at 95°C for 2 h. If the time is too short, the temperature is too low, and the reaction may not be complete, resulting in agglomeration of PdCuAu NPs (see Figures S5, S6). By comparison, the fluorescence intensity of CuAu, PdAu, PdCu NPs are weak and the products tended to aggregate (see Figures S7A, S8). If there is no Br^−^ in the reaction, irregular and agglomerated PdCuAu NPs can be obtained, which fully shows that Br- plays a key role in the formation of PdCuAu NPs (see Figures S7B, S9). In addition, Cu^2+^ plays a very important role in controlling the morphology and fluorescence of PdCuAu NPs. For bimetallic PdAu, if Cu^2+^ is not added, the resulting nanoparticles are irregular, and these nanoparticles were exhibited weak fluorescence intensity (see Figures S7B, S9). When PVP is not added in the preparation process, many large particles will be obtained and serious agglomeration will occur. Copolymer PVP as a template can effectively improve the dispersion of nucleated nanoparticles and reduce the possibility of agglomeration (see Figures S6B, S8). Moreover, we also found that HCl has a significant effect on the morphology and fluorescence of PdCuAu NPs. In the absence of HCl, agglomerated and irregular nanoparticles were formed due to their rapid reduction kinetics (see Figures S7B,C, S10). Because HCl can reduce the reduction ability of ascorbic acid, the reduction rate of metal salt precursors in the reactants will be reduced when HCl is added to the reaction system. According to the above control experiments, we synthesized the PdCuAu NPs in a water bath at 95°C for 2 h with a molar ratio of Pd/Cu/Au equal to 1:5:1. The optical stability of PdCuAu NPs was further studied by fluorescence spectroscopy, as shown in Figure S11. With the passage of time, the fluorescence intensity of PdCuAu NPs was monitored periodically by fluorescence characterization. The results showed that the fluorescence intensity of PdCuAu NPs changed little within 45 days. Therefore, PdCuAu NPs have good stability.

### Fluorescence Detection for Temperature Sensor

**Figure 4:**
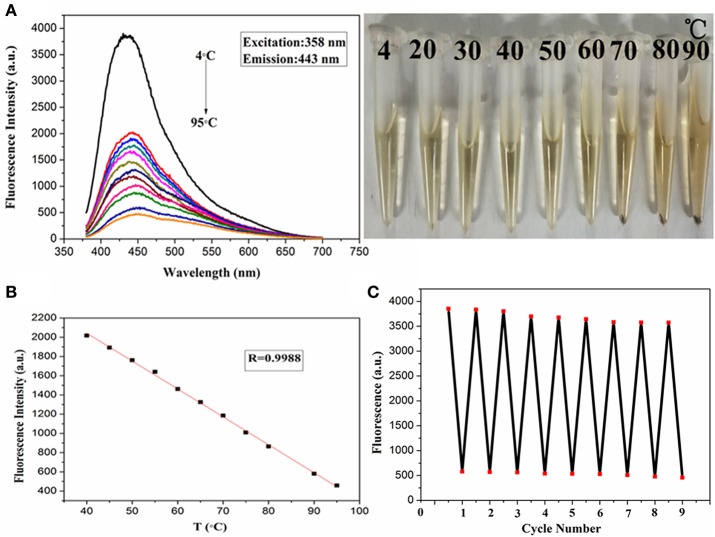
**(A)** Fluorescence spectra of PdCuAu NPs with temperature ranging from 4 to 95°C. **(B)** The linear relationship between changes of temperature and fluorescence intensity. **(C)** The fluorescence response of eight cycles at 4–95°C.

In recent years, there is little research on temperature sensors, and the PdCuAu NPs prepared in this paper have a good response by fluorescence detection in a wide temperature range of 4–95°C. Therefore, PdCuAu NPs has potential application as a temperature sensor. As shown in Figure 4A. The fluorescence intensity of PdCuAu NPs decreased with the increase of temperature, but the emission peak position of PdCuAu NPs did not shift in the temperature range studied. The reason for this phenomenon may be due to the thermal motion of the non-radiative process, that is to say, the frequency of collision and the rate of non-radiative transition of the molecule will increase at high temperature, thus reducing the emission intensity of the excited state (Wang C. et al., 2015; Wang et al., 2016; Jiang et al., 2017). In the temperature range of 4–95°C, the linear relationship between fluorescence intensity and temperature is illustrated in Figure 4B, the linear equation was *F* = −29.03T + 3,204 (*R* = 0.9988). Furthermore, the recyclability and reversibility of resultant PdCuAu NPs based on a fluorescent thermometer were also investigated. As revealed in Figure 4C, the response of PdCuAu NPs to temperature is reversible. After heating and cooling, the reversible process can repeat at least eight cycles without obvious change of fluorescence signal. The results show that PdCuAu NPs have excellent reutilization in response to temperature changes.

### Peroxidase-Like Catalytic Activity of PdCuAu NPs

**Figure 5:**
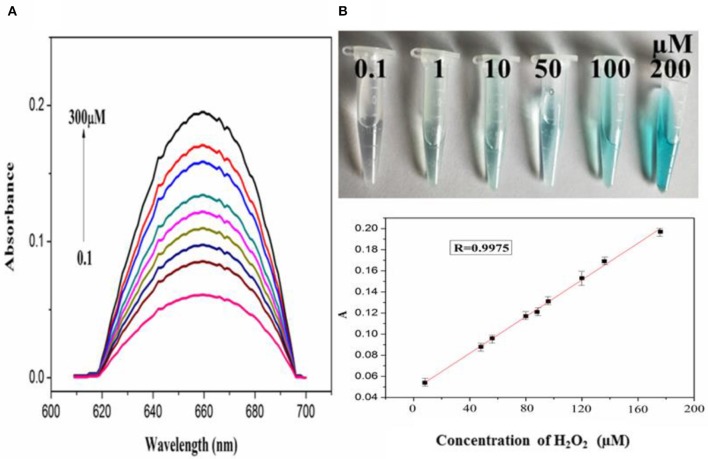
**(A)** The absorption spectra of PdCuAu NPs and TMB system upon adding different concentrations of H_2_O_2_ (0.1–300 μM, from bottom to top). **(B)** The corresponding linear calibration plots for H_2_O_2_, top: the corresponding color changes.

**Figure 6:**
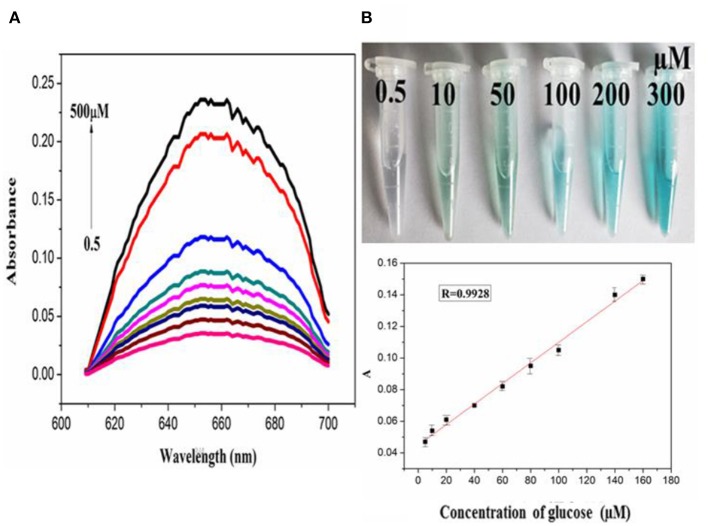
**(A)** The absorption spectra of PdCuAu NPs and TMB system upon adding different concentrations of glucose (0.5–500 μM, from bottom to top). **(B)** The corresponding linear calibration plots for glucose, top: the corresponding color changes.

In order to explore the catalytic activity of PdCuAu NPs, the peroxidase activity of PdCuAu NPs was studied with TMB as chromogenic substrate. The top of Figure 5B point out the color changes in different environments. When there is H_2_O_2_ in the reaction system, PdCuAu NPs can catalyze the oxidation of TMB to produce typical blue products visible to the naked eye, indicating that PdCuAu NPs have the peroxidase activity. Like natural peroxidase HRP, temperature and pH are important factors affecting the catalytic activity of PdCuAu NPs. The peroxidase-like activity of PdCuAu NPs at different pH environments (3.5–6.0) and temperatures (25–60°C) was studied (see Figure S12). The catalytic activity of PdCuAu NPs increases with the increase of pH. When the pH is equal to 5.0, the catalytic activity of PdCuAu NPs reaches the maximum. If the pH < 5.0, the catalytic activity of PdCuAu NPs decreases, this is because pH < 2 easily produces yellow diimine, and pH > 5 will accelerate the decomposition of hydrogen peroxide (see Figure S12A). Besides, the effect of temperature on PdCuAu NPs also discussed, because temperature can accelerate enzymatic reaction, but too high temperature will lead to inactivation of enzymatic reaction, so there is an optimum temperature for enzymatic reaction. The optimum temperature for enzymatic reaction in this study is 40°C (see Figure S12B). For the comparative experiment of catalytic activity of PdCuAu NPs, the steady-state kinetics method was used in this experiment. A typical Michaelis-Menten curve was obtained by controlling the concentration of one peroxidase substrate unchanged and then changing the concentration of another peroxidase substrate (see Figures S13A–D). In Table S1, we can see the kinetic parameters of enzymes derived from Lineweaver-Burk. As everyone knows that K_m_ can express the affinity of specific enzymes to substrates. When the K_m_ value is small, the affinity between enzyme and substrate is strong, whereas a weaker affinity. It can be drawn from Table S1, the K_m_ value of PdCuAu NPs with H_2_O_2_ as the substrate was low. On the one hand, the K_m_ value of PdCuAu NPs with TMB as substrate was low, which indicates that PdCuAu NPs have strong affinity with TMB. In addition, the K_m_ of PdCuAu NPs as shown in Table S1 is lower than that of other reported materials. Therefore, in subsequent experiments, we chose pH = 5.0 and temperature was 40°C as the best reaction conditions. Because PdCuAu NPs have excellent catalytic performance, we designed a convenient, rapid and direct colorimetric method for the detection of H_2_O_2_. Figures 5A,B showed that the absorbance of TMB is positively correlated with the concentration of H_2_O_2_ at 652 nm with the *R* = 0.9975. The linear regression equation obtained was *A* = 6.95 × 10^−3^ [H_2_O_2_] + 0.04713 with a linear range of 0.1–300 μM. When the signal-to-noise ratio (S/N) is 3, the calculated LOD = 5 nM, which is much lower than the detection limit of other reported nanomaterials, for instance Co_3_O_4_ NPs (Ding et al., 2017) GQDs/CuO nanocomposites (Zhang et al., 2017b). GOx can catalyze the oxidation of glucose to produce H_2_O_2_, that is to say, PdCuAu NPs as a peroxidase coupled with GOx to simulate the above-mentioned TMB-H_2_O_2_ system to indirectly detect glucose (see Scheme 1). As shown in Figure 6A, when the maximum absorption wavelength is 652 nm, the absorbance increases with the increase of glucose concentration. The linear range of the standard curve of absorbance changes with glucose concentration is 0.5–500 μM with the *R* = 0.9928 and the linear regression equation obtained was *A* = 6.512 × 10^−4^ [glucose] + 0.04484. When the signal-to-noise ratio (S/N) is 3, the LOD of this method is as low as 25 nM, which is much lower than the LOD reported in the previous literature, such as Pt nanoclusters (Jin et al., 2017), NiCo_2_O_4_/3DGF (Wu et al., 2015). The color change of different concentration of glucose solution can be observed with eyes very clearly (top of Figure 6B). PdCuAu NPs detection system shows a wide linear range and it has excellent sensitivity to glucose. The selectivity of glucose/GOx/PdCuAu NPs/TMB system was also studied. under the same conditions, we selected several other sugars (fructose, sucrose, lactose and maltose) for the comparative experiment. We can get it from Figure S14, glucose analogs had little interference in the detection of glucose. This indicates that the system of glucose/GOx/PdCuAu NPs/TMB has high selectivity for the detection of glucose due to the specificity of GOx. Therefore, glucose sensor based on PdCuAu NPs can be established successfully.

## Conclusions

In conclusion, the synthesis of polymetallic nanomaterials requires very stringent conditions. This is because it is difficult to control the reduction and nucleation process of metal salt precursors in the reactants, so we successfully prepared PdCuAu NPs by a very simple one-pot synthesis method in this experiment. The method is simple in operation, mild in reaction conditions and does not require multi-step synthesis. Moreover, the growth mechanism of PdCuAu NPs was also studied through a series of control experiments. The resultant PdCuAu NPs show remarkable features including fluorescence properties and superior catalytic activity. The research shows that it has good sensitivity to temperature change and good linear relationship, so it can be used in thermal imaging of biological environment. Additionally, the prepared PdCuAu NPs have peroxidase-like catalytic properties. It can catalyze TMB in the presence of H_2_O_2_ to produce color reaction, and the whole experimental process only takes 10 min. Therefore, we constructed a colorimetric sensor with high selectivity and rapid response using PdCuAu NPs as mimetic enzymes, and applied it to the detection of H_2_O_2_ and glucose. The synthesized PdCuAu NPs have excellent temperature sensors and catalytic performance for peroxidase-like enzymes. The PdCuAu NPs have good stability. However, It is not clear which metal of the three metal system has peroxidase properties. According to these studies, this kind of PdCuAu NPs-based sensor has a promising prospect in the biological chemistry.

## Data Availability Statement

All datasets generated for this study are included in the article/Supplementary Material.

## Author Contributions

FN, LG, JA, and YW conceived and carried out experiments, analyzed data, and wrote the paper. All authors read and approved the final manuscript.

### Conflict of Interest

The authors declare that the research was conducted in the absence of any commercial or financial relationships that could be construed as a potential conflict of interest.

## References

[B1] Chen D. Sun P. Liu H. Yang J. (2017). Bimetallic Cu–Pd alloy multipods and their highly electrocatalytic performance for formic acid oxidation and oxygen reduction. J. Mater. Chem. A 5, 4421–4429. 10.1039/C6TA10476B

[B2] Chen M. Ding Y. Gao Y. Zhu X. Wang P. Shi Z. (2017). N, N′-di-caboxy methyl perylene diimides (PDI) functionalized CuO nanocomposites with enhanced peroxidase-like activity and their application in visual biosensing of H_2_O_2_ and glucose. RSC Adv. 7, 25220–25228. 10.1039/C7RA04463A

[B3] Chen R. Xu W. Xiong C. Zhou X. Xiong S. Nie Z.. (2012). High-salt-tolerance matrix for facile detection of glucose in rat brain Microdialysates by MALDI mass spectrometry. Anal. Chem. 84, 465–469. 10.1021/ac202438a 22111575

[B4] Cho Y.-H. Kim O.-H. Chung D. Y. Choe H. Cho Y.-H. Sung Y.-E. (2014). PtPdCo ternary electrocatalyst for methanol tolerant oxygen reduction reaction in direct methanol fuel cell. Appl. Catal. B Environ. 154–155, 309–315. 10.1016/j.apcatb.2014.02.016

[B5] Ding Y. Chen M. Wu K. Chen M. Sun L. Liu Z.. (2017). High-performance peroxidase mimics for rapid colorimetric detection of H_2_O_2_ and glucose derived from perylene diimides functionalized Co_3_O_4_ nanoparticles. Mater. Sci. Eng. C 80, 558–565. 10.1016/j.msec.2017.06.020 28866201

[B6] Dubau L. Asset T. Chattot R. Bonnaud C. Vanpeene V. Nelayah J. (2015). Tuning the performance and the stability of porous hollow PtNi/C nanostructures for the oxygen reduction reaction. ACS Catal. 5, 5333–5341. 10.1021/acscatal.5b01248

[B7] Ge J. Yang X. Luo J. Ma J. Zou Y. Li J. (2019). Ordered mesoporous CoO/CeO2 heterostructures with highly crystallized walls and enhanced peroxidase-like bioactivity. Appl. Mater. Today 15, 482–493. 10.1016/j.apmt.2019.03.009

[B8] Guo J. Wang Y. Zhao M. (2018). 3D flower-like ferrous(II) phosphate nanostructures as peroxidase mimetics for sensitive colorimetric detection of hydrogen peroxide and glucose at nanomolar level. Talanta 182, 230–240. 10.1016/j.talanta.2018.01.080 29501146

[B9] Guo S. Zhang X. Zhu W. He K. Su D. Mendoza-Garcia A.. (2014). Nanocatalyst superior to Pt for oxygen reduction reactions: the case of core/shell Ag(Au)/CuPd nanoparticles. J. Am. Chem. Soc. 136, 15026–15033. 10.1021/ja508256g 25279704

[B10] Han L. Zeng L. Wei M. Li C. Liu A. (2015). Mesoporous carbon composite with novel per-oxidase-like activity towards glucose colorimetric assay. Nano 7, 11678–11685. 10.1039/C5NR02694F 26099042

[B11] Hu A. L. Liu Y. H. Deng H. H. Hong G. L. Liu A. L. Lin X. H.. (2014). Fluorescent hydrogen peroxide sensor based on cupric oxide nanoparticles and its application for glucose and L-lactate detection. Biosens. Bioelectron. 61, 374–378. 10.1016/j.bios.2014.05.048 24912038

[B12] Huang X. Q. Zhao Z. P. Chen Y. Zhu E. B. Li M. F. Duan X. F. (2014). A rational design of carbon-supported dispersive Pt-based octahedra as efficient oxygen reduction reaction catalysts. Energy Environ. Sci. 7, 2957–2962. 10.1039/C4EE01082E

[B13] Hwang S. J. Yoo S. J. Jang S. Lim T.-H. Hong S. A. Kim S.-K. (2011). Ternary Pt–Fe–Co alloy electrocatalysts prepared by electrodeposition: elucidating the roles of Fe and Co in the oxygen reduction reaction. J. Phys. Chem. C 115, 2483–2488. 10.1021/jp106947q

[B14] Jiang G. Zhu H. Zhang X. Shen B. Wu L. Zhang S.. (2015). Core/Shell face-centered tetragonal FePd/Pd nanoparticles as an efficient non-Pt catalyst for the oxygen reduction reaction. ACS Nano 9, 11014–11022. 10.1021/acsnano.5b04361 26434498

[B15] Jiang K. Wang P. Guo S. Zhang X. Shen X. Lu G.. (2016). Ordered PdCu-based nanoparticles as bifunctional oxygen-reduction and ethanol-oxidation electrocatalysts. Angew. Chem. Int. Ed. 55, 9030–9035. 10.1002/anie.201603022 27253520

[B16] Jiang K. Wu J. Wu Q. Wang X. Wang C. Li Y. (2017). Stable fluorescence of green-emitting carbon nanodots as a potential nanothermometer in biological media. Part. Part. Syst. Charact. 34:1600197 10.1002/ppsc.201600197

[B17] Jin L. Meng Z. Zhang Y. Cai S. Zhang Z. Li C.. (2017). Ultrasmall Pt nanoclusters as robust peroxidase mimics for colorimetric detection of glucose in human serum. ACS Appl. Mater. Interfaces 9, 10027–10033. 10.1021/acsami.7b01616 28244734

[B18] Khan M. Yousaf A. B. Chen M. M. Wei C. S. Wu X. B. Huang N. D. (2015). Mixed-phase Pd–Pt bimetallic alloy on graphene oxide with high activity for electrocatalytic applications. J. Power Sources 282, 520–528. 10.1016/j.jpowsour.2015.02.090

[B19] Li C Wang H Li Y Yu H Yin S Xue H.. (2018). Tri-metallic PtPdAu mesoporous nanoelectrocatalysts. Nanotechnology 29, 255404–255413. 10.1088/1361-6528/aabb47 29611816

[B20] Li B. S. Chan S. H. (2013). PtFeNi tri-metallic alloy nanoparticles as electrocatalyst for oxygen reduction reaction in proton exchange membrane fuel cells with ultra-low Pt loading. Int. J. Hydrog. Energ. 38, 3338–3345. 10.1016/j.ijhydene.2013.01.049

[B21] Liu Q. Chen P. Xu Z. Chen M. Ding Y. Yue K. (2017b). A facile strategy to prepare porphyrin functionalized ZnS nanoparticles and their peroxidase-like catalyty for colorimetric sensor of hydrogen peroxide and glucose. Sens. Actuat. B Chem. 251, 339–348. 10.1016/j.snb.2017.05.069

[B22] Liu Q. Jia Q. Zhu R. Shao Q. Wang D. Cui P. (2014). 5,10,15,20-Tetrakis(4-carboxylphenyl)porphyrin–CdS nanocomposites with intrinsic peroxidase-like activity for glucose colorimetric detection. Mater. Sci. Eng. C 42, 177–184. 10.1016/j.msec.2014.05.019 25063108

[B23] Liu Q. Yang Y. Lv X. Ding Y. Zhang Y. Jing J. (2017a). One-step synthesis of uniform nanoparticles of porphyrin functionalized ceria with promising peroxidase mimetics for H_2_O_2_ and glucose colorimetric detection. Sens. Actuat. B Chem. 240, 726–734. 10.1016/j.snb.2016.09.049

[B24] Liu Q. Y. Yang Y. T. Li H. Zhu R. R. Shao Q. Yang S. G.. (2015). NiO nanoparticles modified with 5, 10, 15, 20-tetrakis (4-carboxylpheyl)-porphyrin: promising peroxidase mimetics for H_2_O_2_ and glucose detection. Biosens. Bioelectron. 64, 147–153. 10.1016/j.bios.2014.08.062 25212068

[B25] Luo F. Lin Y. Zheng L. Lin X. Chi Y. (2015). Encapsulation of hemin in metal–organic frameworks for catalyzing the chemiluminescence reaction of the H_2_O_2_-Luminol system and detecting glucose in the neutral condition. ACS Appl. Mater. Interfaces 7, 11322–11329. 10.1021/acsami.5b01706 25928385

[B26] Luo S. Shen P. K. (2017). Concave platinum–copper octopod nanoframes bounded with multiple high-index facets for efficient electrooxidation catalysis. ACS Nano 11, 11946–11953. 10.1021/acsnano.6b04458 27662184

[B27] Nie F. R. Ga L. Ai J. Wang Y. (2018). Synthesis of highly fluorescent Cu/Au bimetallic nanoclusters and their application in a temperature sensor and fluorescent probe for chromium(III) ions. RSC Adv. 8, 13708–13713. 10.1039/C8RA02118J PMC9079805 35539310

[B28] Niu X. Lan M. Zhao H. Chen C. (2013). Highly sensitive and selective Nonenzymatic detection of glucose using three-dimensional porous nickel nanostructures. Anal. Chem. 85, 3561–3569. 10.1021/ac3030976 23458297

[B29] Oemrawsingh S. S. R. Markešević N. Gwinn E. G. Eliel E. R. Bouwmeester D. (2012). Spectral properties of individual DNA-hosted silver nanoclusters at low temperatures. J. Phys. Chem. C 116, 25568–25575. 10.1021/jp307848t

[B30] Shan X. Y. Chai L. J. Ma J. J. Qian Z. S. Chen J. R. Feng H. (2014). B-doped carbon quantum dots as a sensitive fluorescence probefor hydrogenperoxide andglucose detection. Analyst 139, 2322–2325. 10.1039/C3AN02222F 24695439

[B31] Song P. Liu L. Wang A.-J. Zhang X. Zhou S.-Y. Feng J.-J. (2015). One-pot synthesis of platinum–palladium–cobalt alloyed nanoflowers with enhanced electrocatalytic activity for ethylene glycol oxidation. Electrochim. Acta. 164, 323–329. 10.1016/j.electacta.2015.02.229

[B32] Sriphathoorat R. Wang K. Luo S. P. Tang M. Du H. Y. Du X. W. (2016). Well-defined PtNiCo core–shell nanodendrites with enhanced catalytic performance for methanol oxidation. J. Mater. Chem. A 4, 18015–18021. 10.1039/C6TA07370K

[B33] Sun L. Ding Y. Jiang Y. Liu Q. (2017). Montmorillonite-loaded ceria nanocomposites with superior peroxidase-like activity for rapid colorimetric detection of H_2_O_2_. Sensor. Actuat. B Chem. 239, 848–856. 10.1016/j.snb.2016.08.094

[B34] Tang M. Luo S. P. Wang K. Du H. Y. Rinrada S. Shen P. K. (2018). Simultaneous formation of trimetallic Pt-Ni-Cu excavated rhombic dodecahedrons with enhanced catalytic performance for the methanol oxidation reaction. Nano Res. 11, 4786–4795. 10.1007/s12274-018-2063-3

[B35] Tian L. L. Chen Y. L. Wu S. P. Cai Y. H. Liu H. D. Zhang J. (2017). One-pot synthesis of cubic PtPdCu nanocages with enhanced electrocatalytic activity for reduction of H_2_O_2_. RSC Adv. 7, 34071–34076. 10.1039/C7RA03220J

[B36] Wang C. Lin H. Xu Z. Huang Y. Humphrey M. G. Zhang C. (2016). Tunable carbon-dot-based dual-emission fluorescent nanohybrids for ratiometric optical thermometry in living cells. ACS Appl. Mater. Interfaces 8, 6621–6628. 10.1021/acsami.5b11317 26909643

[B37] Wang C. Ling L. Yao Y. Song Q. (2015). One-step synthesis of fluorescent smart thermo-responsive copper clusters: a potential nanothermometer in living cells. Nano Res. 8, 1975–1986. 10.1007/s12274-015-0707-0

[B38] Wang H. J. Yin S. L. Li Y. H. Yu H. J. Li C. J. Deng K. (2018). One-step fabrication of tri-metallic PdCuAu nanothorn assemblies as an efficient catalyst for oxygen reduction reaction. J. Mater. Chem. A. 6, 3642–3648. 10.1039/C7TA10342E

[B39] Wang X. Choi S. I. Roling L. T. Luo M. Ma C. Zhang L.. (2015). Palladium-platinum core-shell icosahedra with substantially enhanced activity and durability towards oxygen reduction. Nat. Commun. 6:7594. 10.1038/ncomms8594 26133469 PMC4506534

[B40] Wang Z. von dem Bussche A. Kabadi P. K. Kane A. B. Hurt R. H. (2013). Biological and environmental transformations of copper-based nanomaterials. ACS Nano 7, 8715–8727. 10.1021/nn403080y 24032665 PMC3894052

[B41] Wu M. Meng S. Wang Q. Si W. Huang W. Dong X. (2015). Nickel–cobalt oxide decorated three-dimensional graphene as an enzyme mimic for glucose and calcium detection. ACS Appl. Mater. Interfaces 7, 21089–21094. 10.1021/acsami.5b06299 26329273

[B42] Xia B. Y. Wu H. B. Li N. Yan Y. Lou X. W. Wang X. (2015). One-pot synthesis of Pt-Co alloy nanowire assemblies with tunable composition and enhanced electrocatalytic properties. Angew. Chem., Int. Ed. 54, 3797–3801. 10.1002/anie.201411544 25630856

[B43] Xu G.-R. Liu F.-Y. Liua Z.-H. Chen Y. (2015). Ethanol-tolerant polyethyleneimine functionalized palladium nanowires in alkaline media: the “molecular window gauze” induced the selectivity for the oxygen reduction reaction. J. Mater. Chem. A 3, 21083–21089. 10.1039/C5TA06644A

[B44] Xue Q. Xu G. R. Mao R. D. Liu H. M. Zeng J. H. Jiang J. X. (2017). Polyethyleneimine modified AuPd@PdAu alloy nanocrystals as advanced electrocatalysts towards the oxygen reduction reaction. J. Energy Chem. 26, 1153–1159. 10.1016/j.jechem.2017.06.007

[B45] Yan G. Zhang Y. Di W. (2018). An enzymatic reaction mediated glucose sensor activated by MnO_2_ nanosheets acting as an oxidant and catalyst. Analyst 143, 2915–2922. 10.1039/C8AN00657A 29790497

[B46] Yang L. Zhang Q. Cui Z. Gerboth M. Zhao Y. Xu T. T.. (2017). Ballistic phonon penetration depth in amorphous silicon dioxide. Nano Lett. 17, 7218–7225. 10.1021/acs.nanolett.7b02380 29087722

[B47] Yang X. Cheng X. Ma J. Zou Y. Luo W. Deng Y. (2019). Large-pore mesoporous CeO2–ZrO2 solid solutions with in-pore confined Pt nanoparticles for enhanced CO oxidation. Small 15:1903058. 10.1002/smll.201903058 31389182

[B48] Yin A. X. Min X. Q. Zhu W. Liu W. C. Zhang Y. W. Yan C. H. (2012). Pt-Cu and Pt-Pd-Cu concave nanocubes with high-index facets and superior electrocatalytic activity. Chem. Eur. J. 18, 777–782. 10.1002/chem.201102632 22170590

[B49] Yousaf A. B. Imran M. Uwitonze N. Zeb A. Zaidi S. J. Ansari T. M. (2017a). High-temperature and high-pressure pyrolysis of hexadecane: molecular dynamic simulation based on reactive force field (ReaxFF). J. Phys. Chem. C 121, 2069–2078. 10.1021/acs.jpca.6b12367 28248502

[B50] Yousaf A. B. Imran M. Zaidi S. J. Kasak P. Ansari T. M. Manzoor S. (2017b). Synergistic effect of interfacial phenomenon on enhancing catalytic performance of Pd loaded MnOx–CeO_2_-C hetero-nanostructure for hydrogenation and electrochemical reactions. J. Mater. Chem. A 5, 10704–10712. 10.1039/C7TA02122D

[B51] Zhang L. Chen M. Jiang Y. Chen M. Ding Y. Liu Q. (2017a). A facile preparation of montmo rillonite supported copper sulfide nanocomposites and their application in the detection of H_2_O_2_. Sens. Actuat. B Chem. 239, 28–35. 10.1016/j.snb.2016.07.168

[B52] Zhang L. Hai X. Xia C. Chen X. W. Wang J. H. (2017b). Growth of CuO nanoneedles on graphene quantum dots as peroxidase mimics for sensitive colorimetric detection of hydrogen peroxide and glucose. Sens. Actuat. B Chem. 248, 374–384. 10.1016/j.snb.2017.04.011

[B53] Zhao T. T. Chen Q. Y. Yang H. (2015). Spectroscopic study on the formation of DNA-Ag clusters and its application in temperature sensitive vehicles of DOX. Spectrochim. Spectrochim. Acta A Mol. Biomol. Spectrosc. 137, 66–69. 10.1016/j.saa.2014.08.025 25200118

[B54] Zhou W. J. Zhu J. B. Teng Y. Du B. J. Han X. Dong S. J. (2018). Novel dual fluorescence temperature-sensitive chameleon DNA-templated silver nanocluster pair for intracellular thermometry. Nano Res. 11, 2012–2023. 10.1007/s12274-017-1817-7

