# Supplementary material for: Trimetallic PdCuAu Nanoparticles for Temperature Sensing and Fluorescence Detection of H2O2 and Glucose
Source: Front Chem. 2020 Apr 7;8:244. doi: 10.3389/fchem.2020.00244 (PMC7154178; doi:10.3389/fchem.2020.00244)
Supplement: Supplementary file 1 [file Data_Sheet_1.pdf]

Supporting Information

Trimetallic PdCuAu Nanoparticles for Temperature Sensing and Colorimetric Detection of H_2O_2 and Glucose

Furong Nie[§, 1], *Lu Ga*[†, 1], *Jun Ai*[§, *], *Yong Wang*[£, *]

[§] College of Chemistry and Enviromental Science, Inner Mongolia Normal University, 81 Zhaowudalu, Hohhot 010022, China.

[†] *College of Pharmacy, Inner Mongolia Medical University, Jinchuankaifaqu, Hohhot 010110, China.*

[£] College of Geographical Science, Inner Mongolia Normal University, 81 Zhaowudalu, Hohhot 010022, China.

1：These authors contributed equally to this work.

Corresponding Author: Jun Ai, Yong Wang

Affiliation- College of Chemistry and Enviromental Science, Inner Mongolia Normal University

E-mail：imacaj01@163.com；wangyonglsx@163.com

Table of Contents

1. **Figure S1.** TEM-EDS spectrum of the as-prepared PdCuAu NPs (the sample dropwise adding one drop of the suspension on a nickel TEM grid coated with a holey carbon film)……..…..…..……………………………..…………………..……………..S3

2. **Figure S2.** The fluorescence spectra of PdCuAu NPs at different Pd/Cu/Au molar ratios…S4

3. **Figure S3.** The TEM images of PdCuAu NPs at different Pd/Cu/Au molar ratios...…..…...S4

4. **Figure S4.** (a) The fluorescence spectra of PdCuAu NPs at different reaction times. (b) The fluorescence spectra of PdCuAu NPs at different temperatures...………..……..…..…..…...S5

5. **Figure S5.** The TEM images of PdCuAu NPs at different reaction times.……..…..…..…...S5

6. **Figure S6.** The TEM images of PdCuAu NPs at different temperatures……..…..…..…….S6

7. **Figure S7.** (a) The fluorescence spectra of CuAu, PdAu, PdCu and PdCuAu NPs. (b) The fluorescence spectra of PdCuAu NPs and without Cu^{2+}, HCl, KBr, PVP, respectively. (c) The fluorescence spectra of the effect of HCl on PdCuAu NPs……..…..…..…………………..S7

8. **Figure S8.** TEM images of the CuAu, PdAu and PdCu NPs prepared under the identical conditions used for the typical synthesis……..…..…..……………………………………..S7

9. **Figure S9.** TEM images of the samples prepared without KBr, Cu^{2+}, PVP, respectively, under the identical conditions used for the typical synthesis.………………………………S7

10. **Figure S10.** TEM images of the effect of HCl on PdCuAu NPs.…………………………….…………………………….…………………………S8

11. **Figure S11.** Fluorescence spectra of the PdCuAu NPs with time ranging from 5 to 45 day.…..…..…..…………………………..…………………………..………………….....S8

12. **Figure S12.** The effect of pH (a) and temperature (b) on the catalytic activity of PdCuAu NPs.……..…..…..…………………………..…………………….…………………S9

13. **Figure S13.** Steady-state kinetic analysis by the Michaelis-Menten model and catalytic mechanism of the reaction catalyzed by PdCuAu NPs. (a): TMB concentration was fixedcat 1.0 mM and the H_2O_2 concentration was varied. (b): H_2O_2 concentration was fixed at 0.1 mM and the TMB concentration was varied. (c, d): Error bars represent the standard deviaton based on three repeated measurements.……..…..…..……………………….....................S9

14. **Figure S14.** The selectivity of the glucose/GOx/PdCuAu NPs/TMB……..…..…..……..S10

15. **Table S1.** Comparison of the kinetic parameters of PdCuAu NPs with other reported typical enzyme mimics. K_m is the Michaelis-Menten constant, V_{max} is the maximal reaction velocity, [E] is the concentration of nanoparticles and k_{cat} is the catalytic constant, where $k_{cat} = V_{max} / [E]$ ………………………………..……………………..……………..................................S11

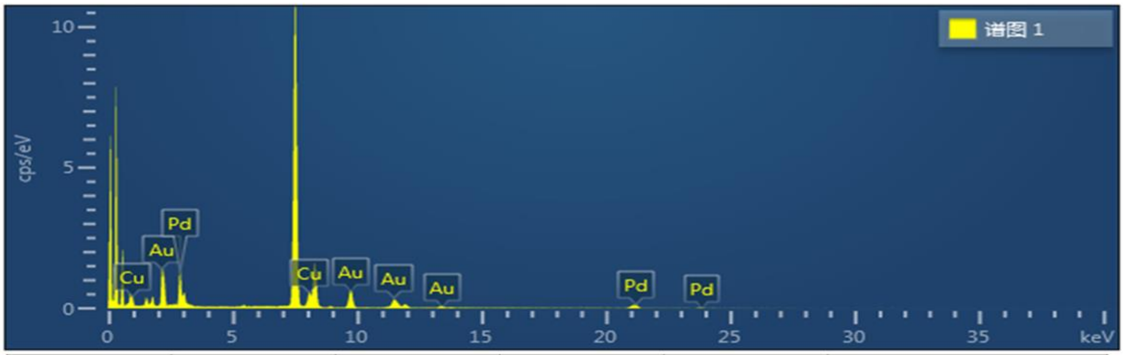

Element	Line type	k factor	Absorption correction	wt%	wt% Sigma
Cu	K line	1.230	1.00	2.24	0.39
Pd	K line	8.900	1.00	42.14	1.23
Au	L line	2.243	1.00	55.62	1.21
Total:				100.00	

Figure S1. TEM-EDS spectrum of the as-prepared PdCuAu NPs (the sample dropwise adding one drop of the suspension on a nickel TEM grid coated with a holey carbon film).

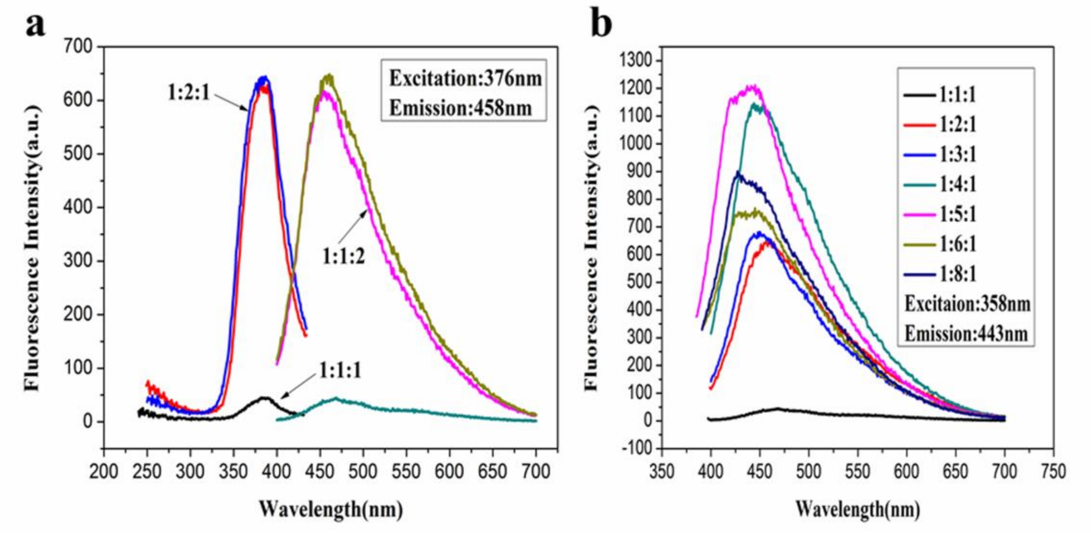

Figure S2. The fluorescence spectra of PdCuAu NPs at different Pd/Cu/Au molar ratios.

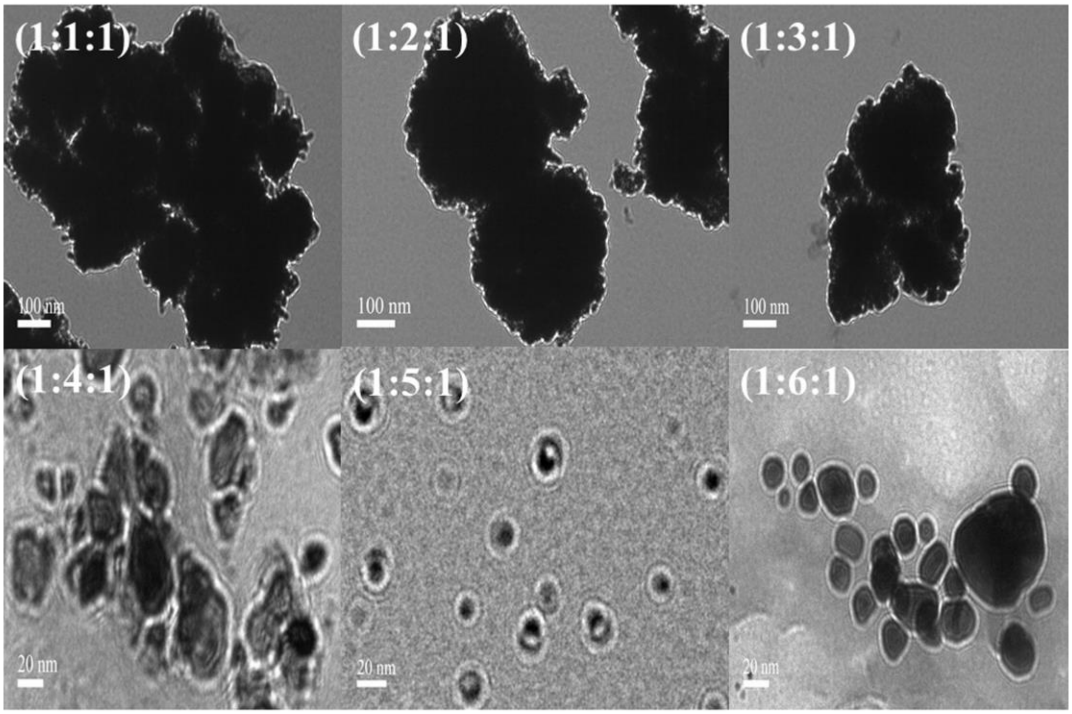

Figure S3. The TEM images of PdCuAu NPs at different Pd/Cu/Au molar ratios.

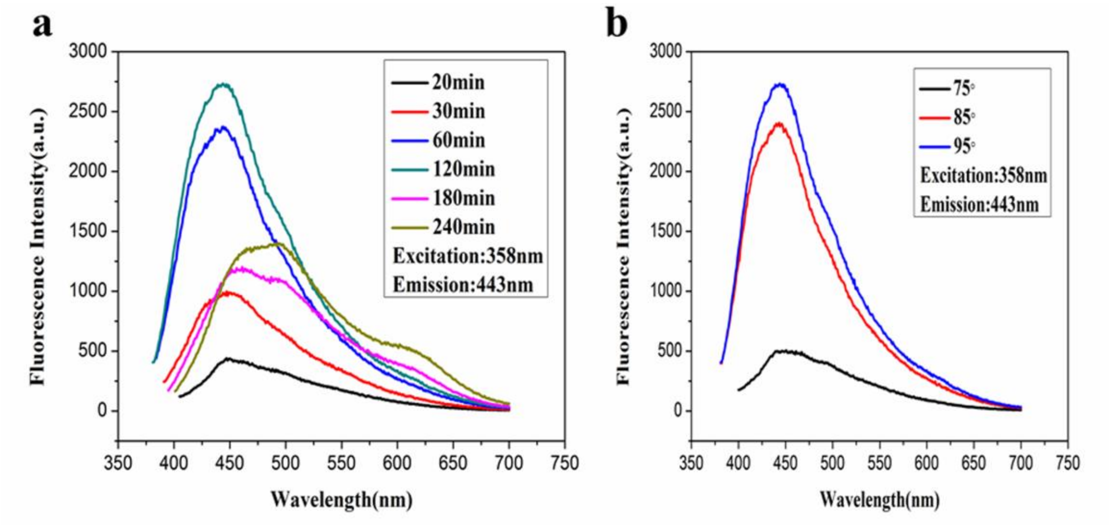

Figure S4. (a) The fluorescence spectra of PdCuAu NPs at different reaction times. (b) The fluorescence spectra of PdCuAu NPs at different temperatures.

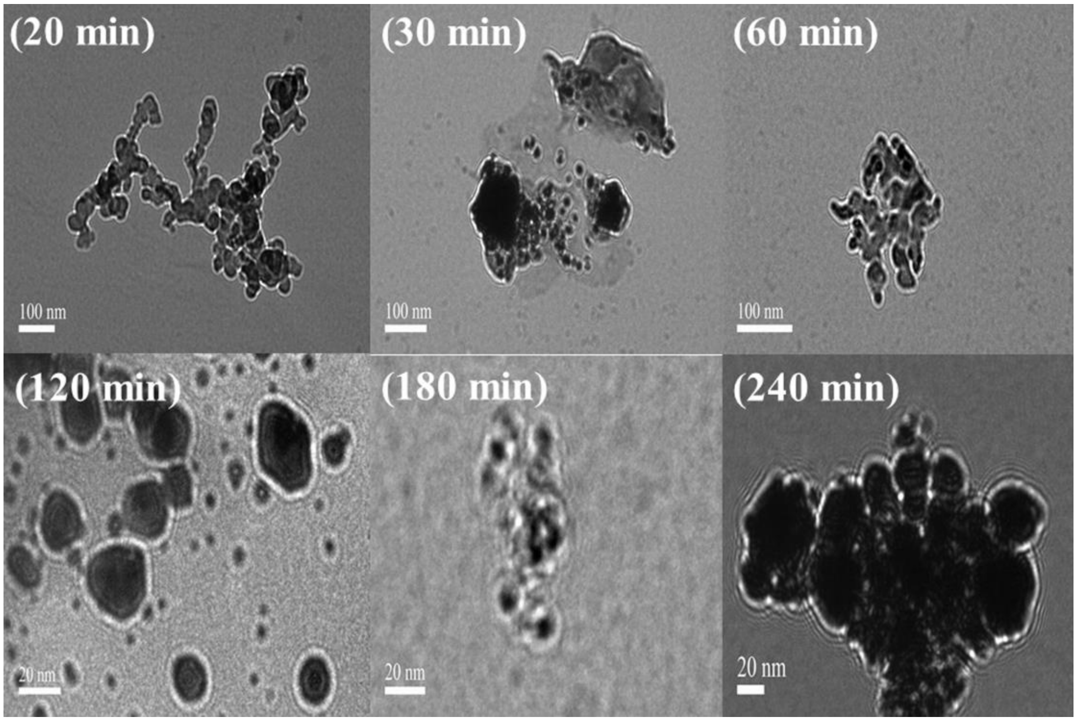

Figure S5. The TEM images of PdCuAu NPs at different reaction times.

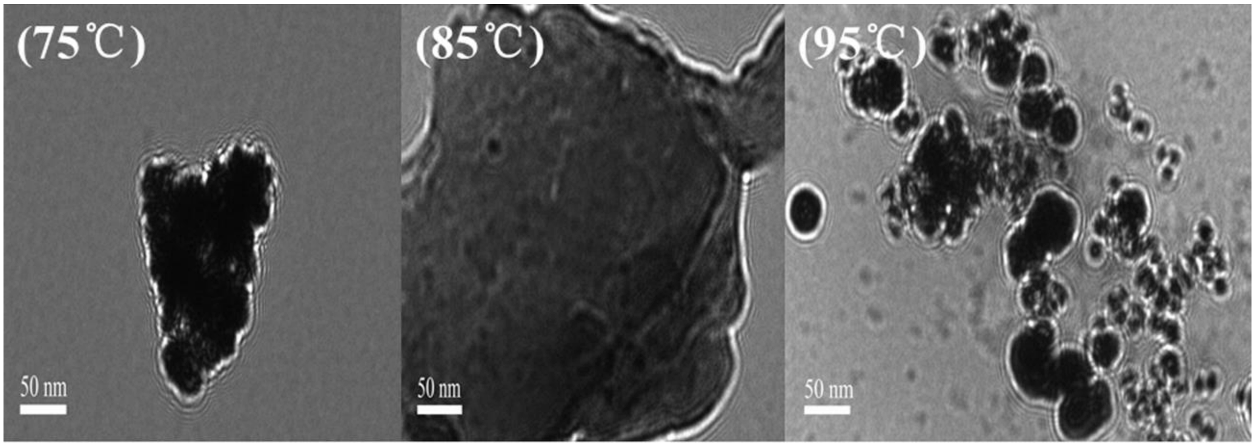

Figure S6. The TEM images of PdCuAu NPs at different temperatures.

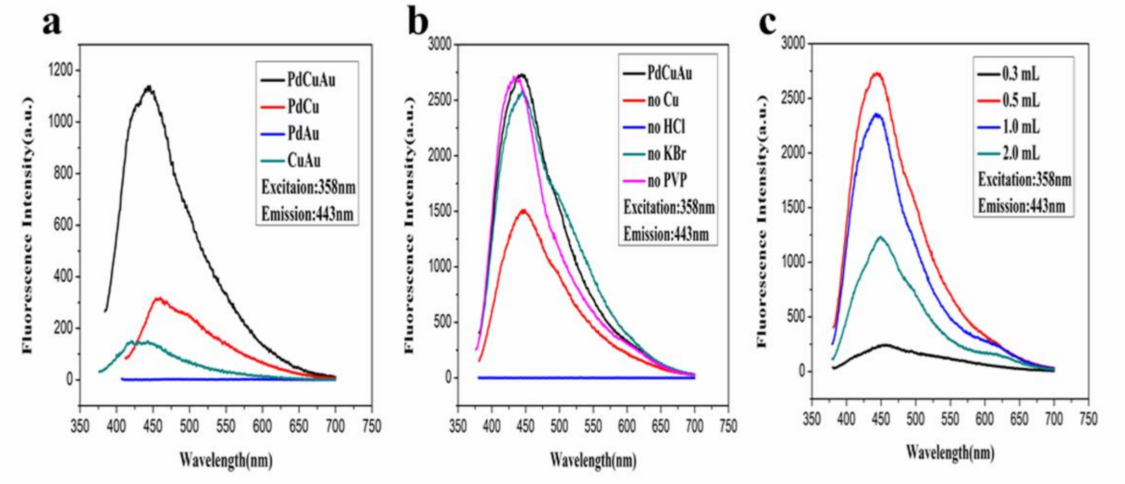

Figure S7. (a) The fluorescence spectra of CuAu, PdAu, PdCu and PdCuAu NPs. (b) The fluorescence spectra of PdCuAu NPs and without Cu^{2+}, HCl, KBr, PVP, respectively. (c) The fluorescence spectra of the effect of HCl on PdCuAu NPs.

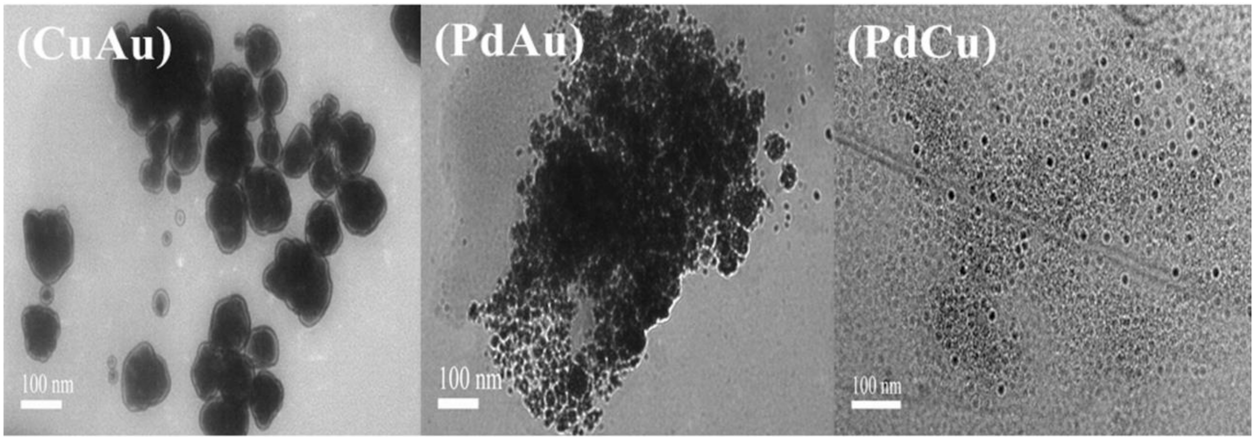

Figure S8. TEM images of the CuAu, PdAu and PdCu NPs prepared under the identical conditions used for the typical synthesis.

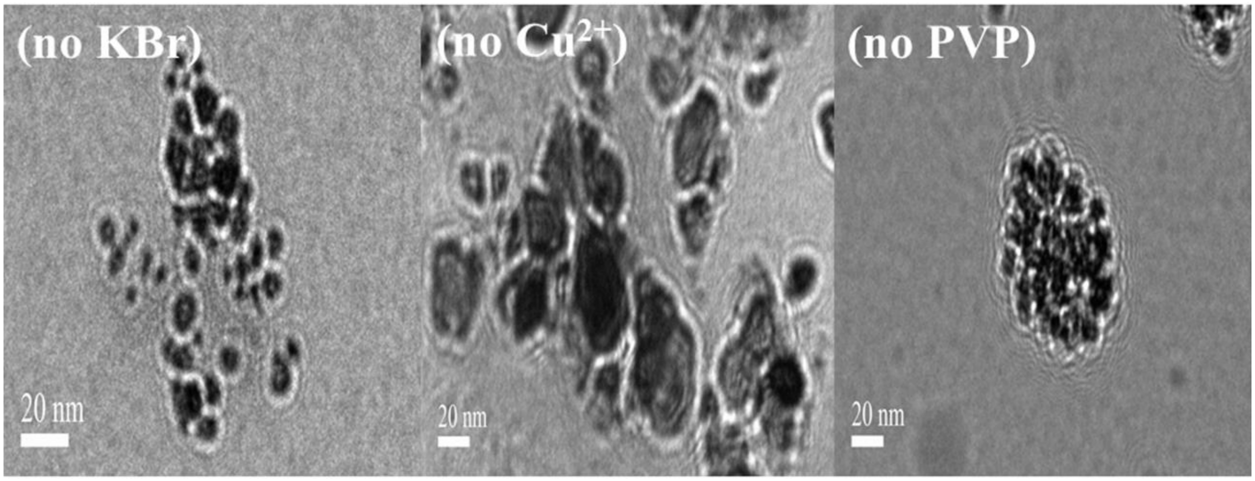

Figure S9. TEM images of the samples prepared without KBr, Cu^{2+}, PVP, respectively, under the identical conditions used for the typical synthesis.

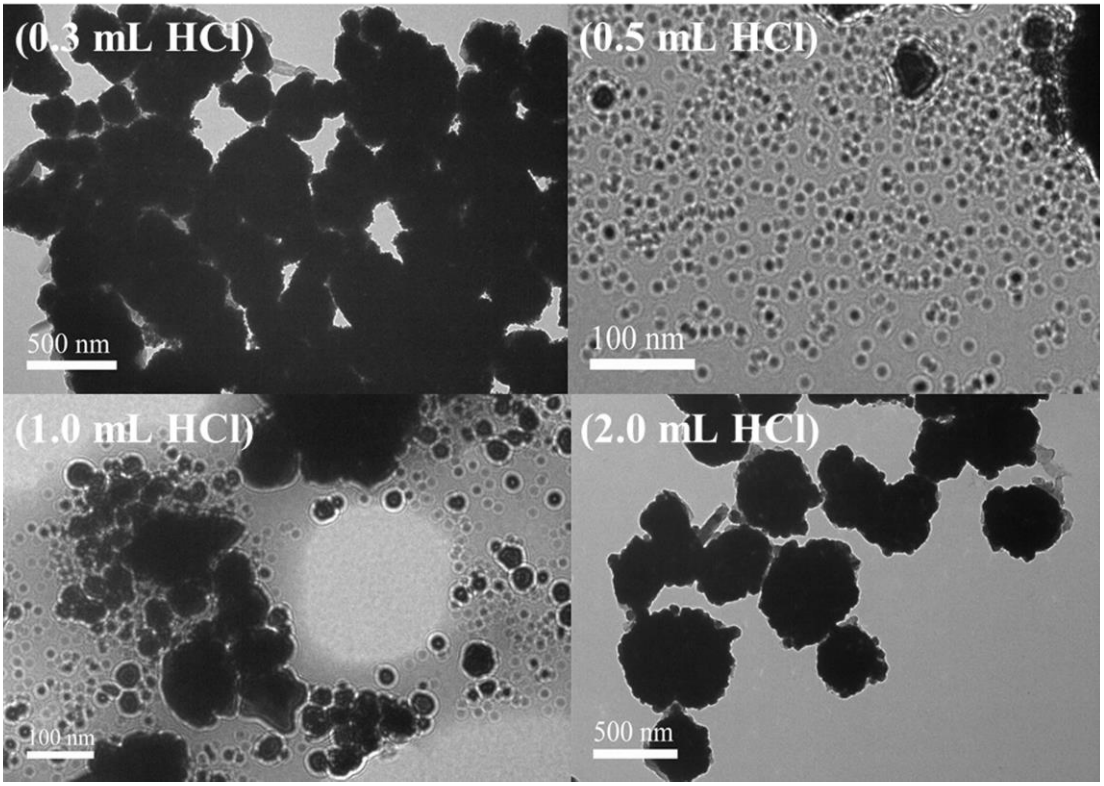

Figure S10. TEM images of the effect of HCl on PdCuAu NPs.

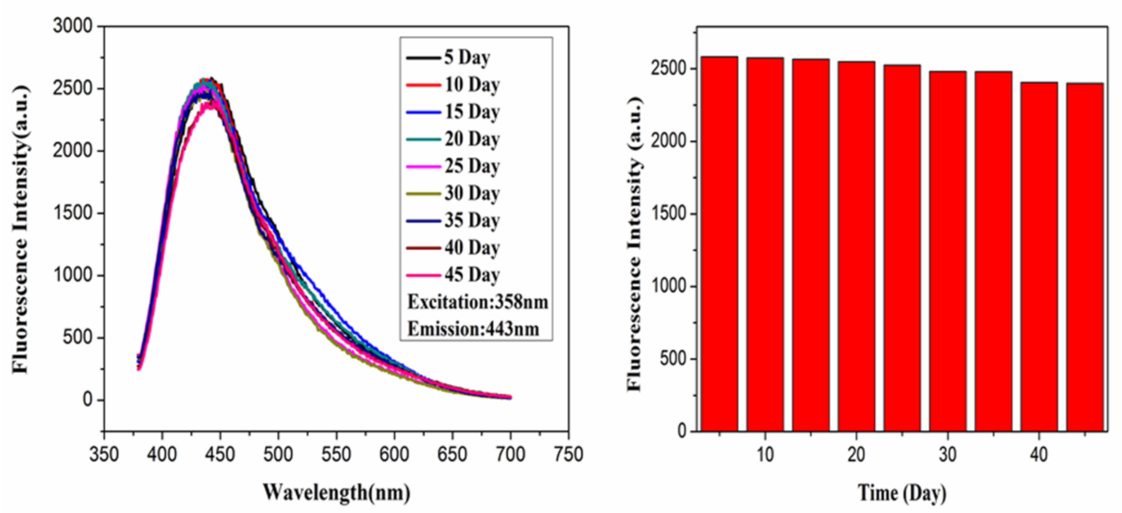

Figure S11. Fluorescence spectra of the PdCuAu NPs with time ranging from 5 to 45 day.

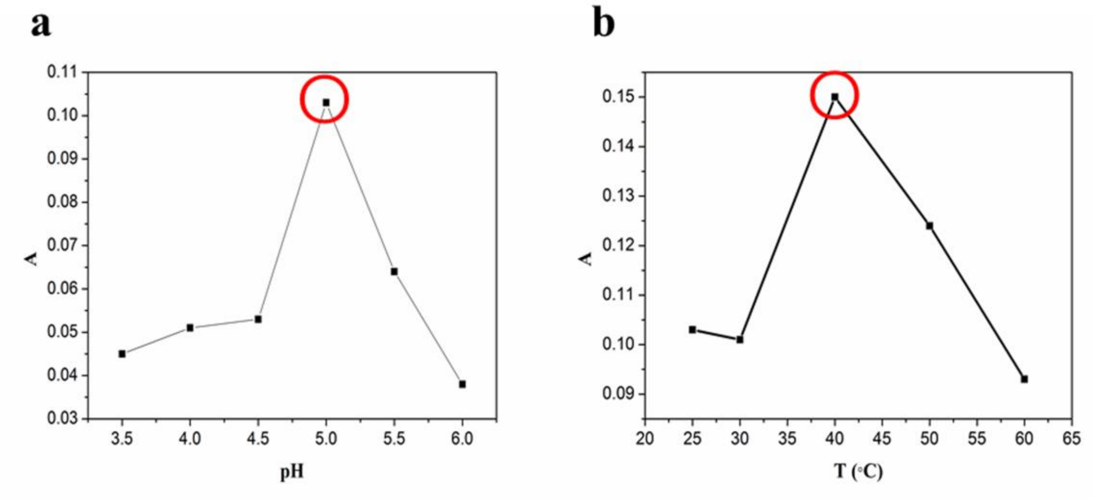

Figure S12. The effect of pH (a) and temperature (b) on the catalytic activity of PdCuAu NPs.

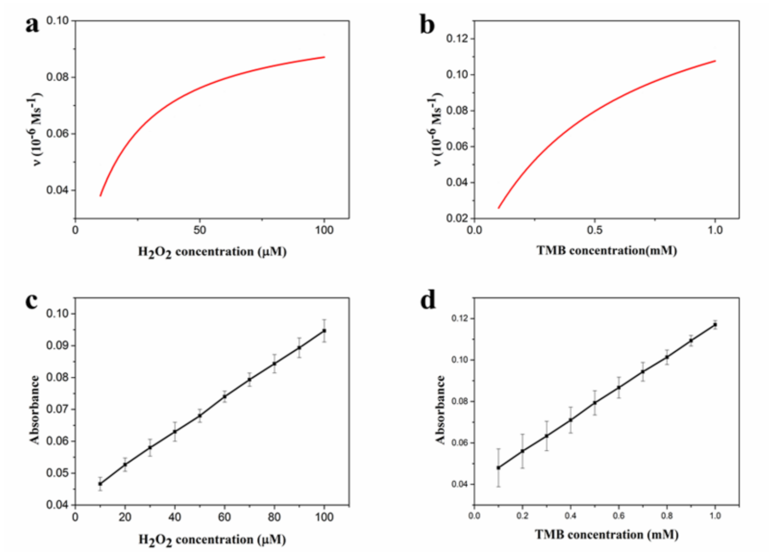

Figure S13. Steady-state kinetic analysis by the Michaelis-Menten model and catalytic mechanism of the reaction catalyzed by PdCuAu NPs. (a): TMB concentration was fixedcat 1.0 mM and the H_2O_2 concentration was varied. (b): H_2O_2 concentration was fixed at 0.1 mM and the TMB concentration was varied. (c, d): Error bars represent the standard deviation based on three repeated measurements.

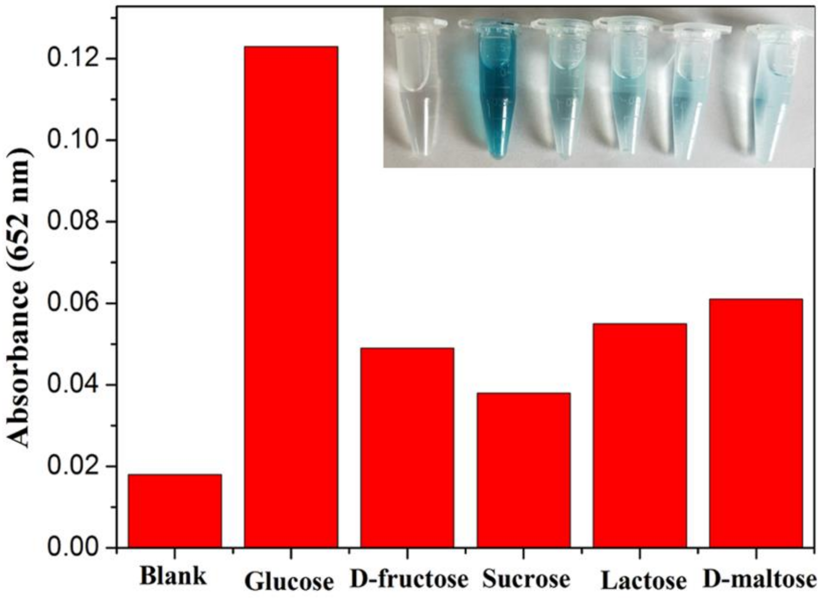

Figure S14. The selectivity of the glucose/GOx/PdCuAu NPs/TMB.

Table S1 Comparison of the kinetic parameters of PdCuAu NPs with other reported typical enzyme mimics. K_m is the Michaelis-Menten constant, V_{max} is the maximal reaction

velocity, [E] is the concentration of nanoparticles and k_{cat} is the catalytic constant, where $k_{cat} = V_{max} / [E]$.

**Catalyst**	**[E] (M)**	**Substrate**	**K_m (mM)**	**V_{max} ($10^{-7}Ms^{-1}$)**	**k_{cat} (s^{-1})**	**Ref**
		H_2O_2	173.51	1.89	0.75×10^2	
Co_3O_4	2.53×10^{-9}	TMB	0.10	2.56	1.01×10^2	[S1]
PVP-Ir		H_2O_2	266	3.85	1.96×10^2	
NPs	1.97×10^{-9}	TMB	0.02	1.08	0.55×10^2	[S2]
		H_2O_2	188	3200	7.55×10^3	
Ft-Pt NPs	4.24×10^{-8}	TMB	0.22	5.58	0.13×10^2	[S3]
		H_2O_2	199.4	0.93	7.20×10^4	
Au-Ft	1.3×10^{-12}	TMB	0.097	0.75	5.80×10^4	[S4]
$Fe_3(PO_4)_2 \cdot 8H_2O$		H_2O_2	0.11	5.58	2.11×10^5	
	2.6×10^{-12}	TMB	0.36	1.58	6.00×10^4	[30]
PdCuAu		H_2O_2	0.16	1.2	2.18×10^4	
NPs	5.5×10^{-12}	TMB	0.54	1.7	3.09×10^4	This work

References

[S1] Mu, J.; Wang, Y.; Zhao, M.; Zhang, L. Intrinsic peroxidase-like activity and catalase-like activity of Co_3O_4 nanoparticles. *Chem. Commun.* **2012**, *48*, 2540–254.

[S2] Su, H.; Liu, D. D.; Zhao, M.; Hu, W. L.; Xue, S. S.; Cao, Q.; Le, X. Y.; Ji, L. N.; Mao, Z. W. Dual-Enzyme Characteristics of Polyvinylpyrrolidone-Capped Iridium Nanoparticles and Their Cellular Protective Effect against H_2O_2-Induced Oxidative Damage. *ACS Appl. Mater. Interfaces* **2015**, *7*, 8233–8242.

[S3] Fan, J.; Yin, J. J.; Ning, B.; Wu, X.; Hu, Y.; Ferrari, M.; Anderson, G. J.; Wei, J.; Zhao, Y.; Nie, G. Direct evidence for catalase and peroxidase activities of ferritin-platinum nanoparticles. *Biomaterials* **2011**, *32*, 1611–1618.

[S4] Jiang, X.; Sun, C.; Guo, Y.; Nie, G.; Xu, L. Peroxidase-like activity of apoferritin paired gold clusters for glucose detection. *Biosens. Bioelectron*. **2015**, *64*, 165–170.
